# Sleep triggered by an immune response in *Drosophila *is regulated by the circadian clock and requires the NFκB Relish

**DOI:** 10.1186/1471-2202-11-17

**Published:** 2010-02-09

**Authors:** Tzu-Hsing Kuo, Douglas H Pike, Zahra Beizaeipour, Julie A Williams

**Affiliations:** 1 Center for Advanced Biotechnology and Medicine, Department of Pharmacology University of Medicine and Dentistry of New Jersey - Robert Wood Johnson Medical School, Piscataway, NJ 08854, USA

## Abstract

**Background:**

Immune challenge impacts behavior in many species. In mammals, this adaptive behavior is often manifested as an increase in sleep. Sleep has therefore been proposed to benefit the host by enhancing immune function and thereby overcome the challenge. To facilitate genetic studies on the relationship between sleep and immune function, we characterized the effect of the immune response on sleep in *Drosophila melanogaster*. Behavioral features of sleep as well as the innate immune response signaling pathways are well characterized in flies and are highly conserved in mammals.

**Results:**

An immune response induced by infection with Gram-negative bacteria or by aseptic injury increased sleep in flies. The increase in sleep occurred during the morning hours after treatment and the magnitude of the effect was dependent on the time-of-day of inoculation or injury such that night-time treatment had a stronger effect than that during the daytime. This pattern persisted in constant darkness, indicating a role of the circadian clock. Mutants of the circadian clock gene, *period*, eliminated the increase in sleep observed in the morning, but instead showed enhanced sleep immediately after injury or infection.

Null mutants of the Nuclear Factor κB (NFκB) Relish, which is central to the innate immune response, do not increase sleep in response to injury or infection at any time of day. Instead, they maintain a normal sleep pattern until they die. Expression of a full-length Relish transgene in the fat bodies of Relish mutants restored the morning increase in sleep during an immune response. Fat bodies are a major site of immune signalling in flies and have a key role in host defense.

**Conclusions:**

These data demonstrate that an immune response increases sleep in flies in a manner that is gated by the circadian clock and that requires the NFκB *Relish*. These findings support a role of sleep in a recovery process and demonstrate a conserved feature of the Drosophila model of sleep.

## Background

Sleepiness and excess sleep are commonly experienced with infectious illness and other diseases, such as cancer [1]. Sleep has been proposed to be an adaptive response to immune challenge, and to thereby have a role in sustaining a robust immune system [2,3]. The immune system and sleep are indeed tightly linked in mammals, as components of the innate immune response, particularly proinflammatory cytokines such as interleukin-1 (IL-1) and tumor necrosis factor α (TNFα), promote sleep likely through their actions in hypothalamic nuclei. Exogenous application of these compounds increases sleep in mammals, depending on the dose, time of day and the site of injection [4]. In addition, loss of either the IL-1 Type I [5] or the 55 kDa TNF [6] receptor in mice reduces baseline (spontaneous, undisturbed) sleep during the light hours or during the dark hours, respectively. Although some of the molecular components of the mammalian immune system have a well defined role in sleep, we lack a full understanding of how these two physiological processes interact. Determining how sleep is involved in the immune response will have important implications for understanding its putative role in a recovery process as well as for human health.

*Drosophila melanogaster *has proven to be a powerful model for studying sleep. Flies exhibit all of the behavioural features of a sleep-like state [7,8]. One example of these features is that keeping flies awake for an extended period results in a compensatory sleep response, which indicates that flies have a need for this behaviour and that it is controlled by a homeostatic mechanism. Key to understanding a function of sleep is to determine its relationship with other physiological processes. Recent studies have indeed demonstrated that sleep in Drosophila is involved in aging [9], learning [10-13], and immune responses [14].

The first indication that sleep and the immune response are linked in Drosophila was the observation that many immune related genes increase mRNA expression after sleep deprivation [14-16]. The innate immune response in Drosophila is highly conserved with that in mammals, as they express central immune signalling components with corresponding functions. For example, the NFκB protein Relish, which is most similar to NFκB2 or p100 in humans [17], is central to the immune deficiency (Imd) pathway in flies, and is sensitive to injury and infection with Gram-negative bacteria [18]. In our previous report, we confirmed that *Relish *gene expression increases with short term sleep deprivation [14], which is consistent with observations that NFκB activity increases in the brain of mouse or rat [19-22] and human blood mononuclear cells [23] after sleep deprivation. We also demonstrated that flies deficient in, but not lacking, *Relish *expression exhibit decreased baseline, or spontaneous undisturbed sleep [14].

To further explore the association between sleep and the immune response, we determined the effect of infection with Gram-negative bacteria and aseptic injury on sleep in Drosophila. We show that the immune response triggered by both infection and injury promotes sleep in flies and that this effect is controlled by the circadian clock. We also show that the NFκB *Relish *has a strong role in promoting sleep during the immune response and that its expression in fat body is required for its effect on sleep. Together, these data suggest a role of sleep in a recovery process during immune challenge and demonstrate a behavioral feature of Drosophila sleep that is shared with that in mammals.

## Results and Discussion

### Infection and injury promote sleep

We monitored sleep in Canton Special (CS) flies during an immune response that was triggered either by infection (inf) with *E. Coli *or by aseptic injury (inj; see methods). A handled control group (HC) was subjected to the same handling such that they were removed from the activity monitors and CO_2 _anesthetized for the same duration, but they were not infected or injured. Flies were generally monitored for three baseline days and received treatment on the fourth day at one of four different time points in a 12: 12 light: dark (LD) cycle: ZT 0, ZT 6, ZT 12 or ZT 18 (ZT = zeitgeber time, where ZT 0-12 = lights on, and ZT 12-24 = lights off). Sleep was monitored for three days after treatment.

**Figure 1:**
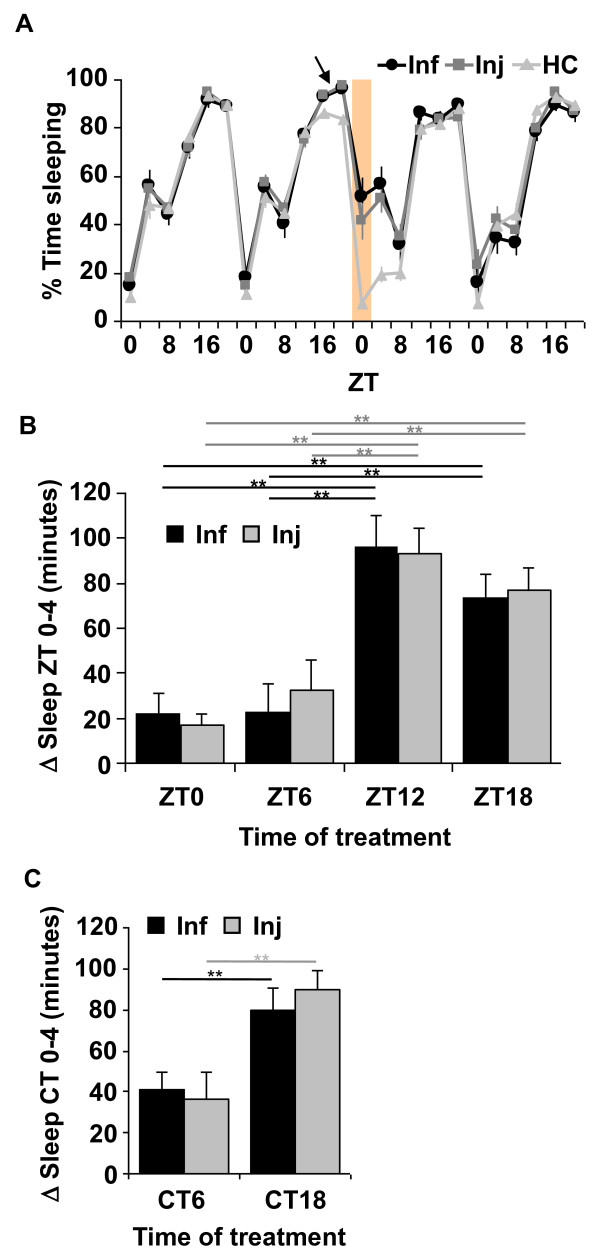
**Infection and injury promote sleep from ZT 0-4 after treatment in wild-type CS flies**. (A) Representative results from infection (Inf) with *E. coli *and aseptic injury (Inj) at ZT 18 (arrow). Mean ± SEM percent time sleeping is plotted for 4 consecutive days in 4 hr increments. Shaded area indicates the increase in sleep from ZT 0-4 after treatment. [n = 12 for Inf; n = 15 for Inj; and n = 16 for handled control group (HC).] (B) Mean ± SEM net changes in sleep are reported from ZT 0-4 the morning after treatment at each of four time points (ZT 0, 6, 12 or 18). *p *< 0.0001, ANOVA; ** p < 0.01, Tukey's post-hoc comparisons within infected or injured groups. n = 26-61 flies per group. (C) Net changes in sleep from circadian time (CT) 0-4, the subjective morning after treatment in constant darkness, at CT 6 or CT 18. ** *p *< 0.01 with student's *t*-test within infected or injured groups. n = 28-32 flies per group. Values for mean net changes in sleep in (B) and (C) and all other subsequent figures, except where otherwise indicated, are normalized to those in handled control groups (see Methods).

Significant increases in sleep were observed in both injured and infected groups relative to HC in the morning hours after treatment. Specifically, the most robust and consistent increase in sleep was from ZT 0-4 (shaded area in Figure 1A), but occasionally lasted up to 8 h (from ZT 0-8). A representative experiment is illustrated in Figure 1A, where flies were treated at ZT 18, and a significant increase in sleep was observed the following morning (shaded area and Additional File 1: Table S1; *p *< 5 × 10^-7^) in both infected and injured groups. Treatment at other times of day also produced increases in sleep the morning after treatment, from ZT 0-4, but with variable effects (Additional File 1: Table S1). In particular, treatment during night time hours produced stronger effects than those in the day time.

We further analyzed the effect of time-of-day of infection and injury on sleep by comparing the net changes in sleep that occurred in the morning from ZT 0-4, after treatment at one of four time points across the day (Figure 1B). To correct for effects of handling, net change in sleep was determined in each fly by calculating the difference in time sleeping in minutes from ZT 0-4 before and after treatment and normalizing to the change in sleep observed between the corresponding time points in the handled control group (see Methods for details). Analysis of variance (ANOVA) revealed a significant effect of time-of-day of treatment in both injured and infected groups (p < 0.0001). Post-hoc comparisons indicated that mean change in sleep from ZT 0-4 that was induced by infection or injury at night, ZT 12 and ZT 18, was significantly larger than sleep induced by that in the daytime, ZT 0 and ZT 6 (Figure 1B).

To ensure that the daily oscillation of the sleep increase associated with the immune response was not an effect of a 12:12 LD cycle, CS flies were infected or injured on the third day in constant darkness (DD). Similar to the effect during LD, both infected and injured flies showed an increase in sleep in the subjective morning hours from CT 0-4 (CT = circadian time) after treatment. The increase in sleep was significantly higher when flies were treated during subjective nighttime (CT 18) than during subjective daytime (CT 6) in both infected and injured groups (Figure 1C).

To exclude the possibility that the increase in sleep was due to an effect on the flies' ability to move by debilitating injury due to the injection, we calculated the average number of activity counts per waking minute (activity rate) for flies treated at ZT 18. We focused on treatment at ZT 18, because this time point produced highly robust effects on sleep as well as on survival rate in response to infection with pathogenic bacteria [24]. If the increase in sleep was due to an effect on the flies' ability to move by injury, we would expect a decrease in activity rate. Instead, waking activity rates were unchanged in the infected group, and higher in injured groups as compared to HC (*p *< 0.05 for ANOVA; *p *< 0.05 for post-hoc comparison in injured and HC group; Additional File 2: Figure S1), indicating that the increase in sleep is not simply a reflection of lack of movement associated with injury.

In summary, an immune response triggered by infection or injury promotes sleep. The increase in sleep is restricted to the morning hours after treatment, and is dependent on the time of day of treatment such that night time treatment produced stronger effects than those in the daytime. This pattern persists in constant darkness, which strongly suggests a role of the circadian clock in regulating this response.

The observation that night-time infection produces a stronger effect on sleep than that in the daytime correlates with a previous observation that infection with pathogenic Gram-negative bacteria at night-time produced a better survival outcome than that in the daytime [24]. Whether the increased sleep experienced by the host contributes to survival during pathogenic infection will be an interesting topic for future study. Interestingly, an earlier study reported a disruption of circadian locomotor rhythm and sleep in flies after infection with Gram-positive pathogenic strains of bacteria [25], which contrasts with our current findings. One possible explanation for this is the different species of bacteria used in each of the studies. The pattern of sleep alteration associated with bacterial infection in mammals is also dependent on the bacterial species and route of infection [26]. Nonetheless, Shirasu-Hiza and colleagues [25] also proposed that in this case, the disruption of circadian rhythms and sleep was a contributing factor to the pathogenesis of the infection, thus further supporting a beneficial role of sleep in host defense.

### Both infection and injury increase NFκB reporter activity

Sleep is a complex process that is sensitive to many different manipulations. It is therefore not surprising that injury and infection produce effects on sleep that are indistinguishable. To ensure the effect was not restricted to *E. coli*, flies were also infected with a mutated Gram-negative bacterial line *P. aeruginosa *(*plcS*) which has pathogenic ability [27]. A similar increase in sleep was observed in the morning from ZT 0-4 after nighttime treatment at ZT 18 in both infected and injured groups (*p *< 5 × 10^-5^; Additional File 3: Figure S2A).

**Figure 2:**
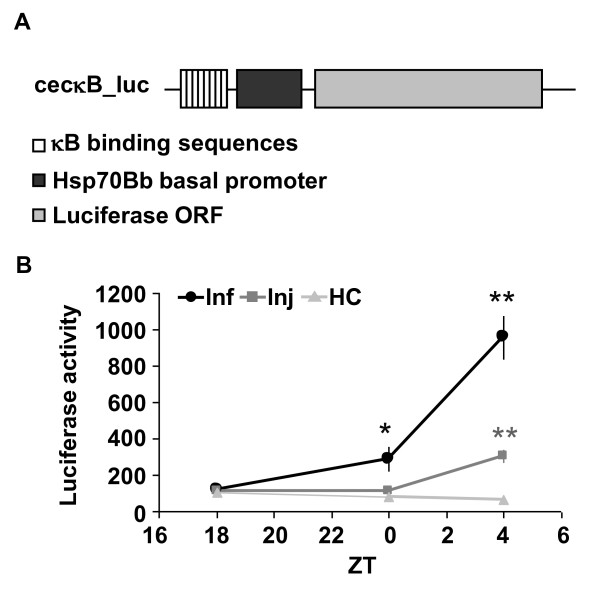
**Infection and injury increase *κB-luc *reporter activity in living flies**. (**A**) The plasmid used for generation of *κB-luc *flies. Plasmid contained 8 copies of κB binding sequences, a heat shock protein (Hsp) 70 Bb basal promoter and a luciferase open reading frame (ORF). (**B**) Mean ± SEM luciferase activity (arbitrary units) was measured at indicated ZT times when *κB-luc *flies were treated at ZT 18. **p < 0.0001 and *p < 0.01, student's *t*-test compared to HC at corresponding time points. n = 32 flies for each group.

Both infection and injury trigger equivalent effects on a cellular immune response [28]. Infection with Gram-negative bacteria, in particular, activates the NFκB *Relish*, which is central to the Imd pathway. Injury also induces Relish-dependent gene expression, but to a lesser extent than infection [29]. To confirm this finding, we measured NFκB dependent luciferase activity in living flies which contain an NFκB response element upstream of a luciferase reporter gene (*κB-luc*; Figure 2A). Both infection with *E. coli *and injury at ZT 18 produced significant increases in *κB-luc *activity by the next morning, at ZT 4, relative to baseline (Figure 2B; ANOVA, *p *< .0001; Tukey's post-hoc comparison, *p *< .01 for both Inf and Inj groups). No significant change in reporter activity was detected in the HC group (ANOVA, *p *< .22). *κB-luc *was increased by approximately 5 fold relative to HC in injured flies, and 15 fold in infected flies. *κB-luc *activity was also sensitive to infection at ZT 18 with *P. aeruginosa*. Large increases in *κB-luc *activity were detected at ZT 4, 10 hours after the infection (*p *< .01, Tukey's post-hoc comparison, relative to baseline at ZT 18), and were further increased at ZT 8 (Additional File 3: Figure S2B). The delay in onset of *κB-luc *activity is consistent with a previous finding that *P. aeruginosa *infection delayed the onset of expression of antimicrobial peptides [30].

These results demonstrate that while infection and injury produce similar effects on sleep, they are clearly distinguishable at a molecular level. The increase in *κB-luc *activity by injury is comparable to that induced by a short term sleep deprivation [14], and both are sufficient to promote sleep (Figure 1, and [14,31]). The observation that infection produces a sustained increase in *κB-luc *activity (Additional File 3: Figure S2B) suggests that a component other than NFκB limits the duration and timing of sleep during immune challenge.

### Sleep during the immune response is disrupted in a clock mutant

To understand why the increase in sleep during an immune response was restricted to the morning hours, we further examined a role of the circadian clock in this process. We measured sleep during the immune response in flies which contain a mutation in the clock gene, *period *(*per*^01 ^[32]). *period *is an integral component of the biological clock in insects and vertebrate species [33]. Sleep-wake activity in *per*^01 ^loss of function mutants fluctuates in LD cycles, but is completely arrhythmic in constant darkness [34].

**Figure 3:**
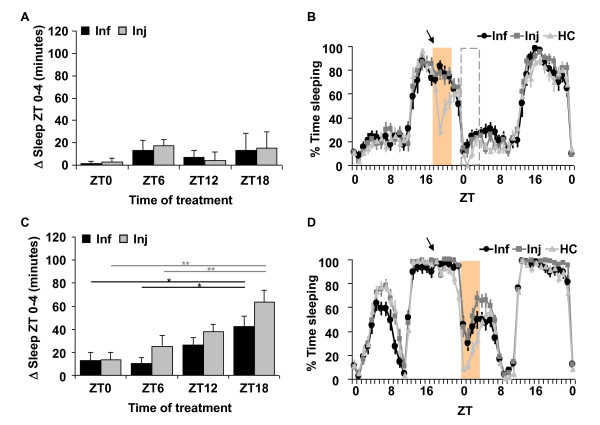
**Sleep during the immune response is gated by the circadian clock**. (A) Mean ± SEM net changes in sleep in *per*^01 ^flies from ZT 0-4 the morning after treatment at each of four time points (ZT 0, 6, 12 or 18). n = 13-16 flies per group. (B) Results from treatment at ZT 18 (arrow) in *per*^01 ^flies. Mean ± SEM percent time sleeping is plotted for 2 consecutive days in 1 hr increments. Shaded area indicates the increase in sleep immediately after treatment from ZT 18-22. Dashed box area highlights ZT 0-4 time and corresponds to results reported in (A); n = 14 for Inf, n = 16 for Inj and n = 13 for HC. (C) Mean ± SEM net changes in sleep in *per*^01^*;;per *flies from ZT 0-4 after treatment at each of four time points (ZT 0, 6, 12 or 18). *p *< 0.0005 and *p *< 0.01 for ANOVA in Inj and Inf groups, respectively. ** *p *< 0.01 and * *p *< 0.05, Tukey's post-hoc comparisons. n = 22-45 flies per group. (D) Representative results from treatment at ZT 18 (arrow) in *per*^01^*;; per *flies. Data are plotted as described in (B). Shaded area indicates the increase in sleep from ZT 0-4 after treatment. n = 14 for Inf, n = 13 for Inj and n = 15 for HC.

Sleep in the morning from ZT 0-4 after infection with *E. coli *or aseptic injury in *per*^01 ^flies was not significantly affected as compared to HC regardless of the time-of-day of treatment (Additional File 1: Table S1 and Figure 3A; ANOVA *p *> .5 for both Inf and Inj groups). Instead, we found that sleep increased immediately after treatment, with the most robust effects occurring within the first four hours (Figure 3B, and Additional File 4: Figure S3A). The immediate increase in sleep in the infected group was significant as compared to HC (*p *< 0.01 for all time points tested; n = 14-16 flies each group) and no significant oscillation across time points was detected (ANOVA, *p *= 0.83). Sleep also increased immediately after injury, but increases were lower when flies were treated during the night time (Additional File 4: Figure S3A; ANOVA *p *< .0001). Figure 3B illustrates an experiment performed in *per*^01 ^mutants treated at night-time, ZT 18. Sleep after manipulation at this time was clearly decreased in the HC group. An earlier study reported that LD cycles mask locomotor behaviour that is otherwise arrhythmic in these flies [34]. The sharp decrease in sleep in the control group is thus attributed to the light exposure that was necessary for administering the injections in the treatment groups. Nonetheless, the flies subjected to infection or injury slept more despite the exposure to light. Wild type CS flies and *per*^01^*;;per *flies, discussed below, did not show this sensitivity to light exposure (compare Figure 3B with Figures 1A and 3D). Handling of *per*^01 ^flies during the daytime period, at ZT 0 and 6, did not produce any changes in sleep in the control group, while sleep in flies subjected to infection or injury at these times produced significant increases in sleep that occurred immediately after the treatment (Additional File 4: Figure S3A).

To confirm the role of *period *in the sleep promoting effect, we examined sleep behaviour during the immune response in *per*^01 ^flies expressing a full-length genomic construct of the *period *gene (*per*^01^*;;per*), as previously described [35]. Locomotor activity rhythms are restored in *per*^01 ^flies carrying this transgene [35,36]. As compared to HC groups, *per*^01^*;;per *flies showed significant increases in sleep in the morning from ZT 0-4 after injury at ZT 6, ZT 12, and ZT 18 (Additional File 1: Table S1). Infection with *E. coli *also produced a significant increase in morning sleep relative to HC, but only when flies were infected at ZT 18 (*p *< .05). In both infected and injured groups, the time-of-day effect on the morning increase in sleep was restored in the *per*^01^*;;per *flies (ANOVA p < 0.0005 for injured, and p < 0.01 for infected groups). Night-time treatment produced stronger effects on morning sleep than that in the daytime (Figure 3C and 3D). In contrast to *per*^01 ^mutants, the immediate effect on sleep occurred only when *per*^01^*;;per *flies were treated at ZT 0 (p < 0.05 for Inf; p < 0.01 for Inj, Additional File 4: Figure S3B). Interestingly, CS flies also exhibited a strong immediate effect on sleep after infection (101 ± 7 minutes, *p *< 1 × 10^-7^) and injury (111 ± 5 minutes, *p *< 5 × 10^-7^) at ZT 0, but not at ZT 6 or at ZT 18. Treatment at ZT 12 produced weaker effects on sleep from ZT 12-16 with an increase of 53 ± 8 minutes (*p *< 0.05) in injured flies. Infected flies also increased sleep at this time, but the increase fell short of significance (*p *< .07).

Together, these data demonstrate a role of the circadian clock in an adaptive behavioural response to bacterial infection and injury. The effect on sleep is gated by the clock, such that the response is restricted to early morning hours. Thus the clock permits a response when flies are subjected to immune challenge at the onset of this period (ZT 0) or at earlier times during the night before (ZT 12 and ZT 18). However, this permissive gate is 'closed' shortly after the morning period, and flies do not increase sleep when challenged at ZT 6. As discussed above, this gating pattern persists in constant dark conditions (Figure 1C) and is abolished in *per*^01 ^mutants, which lack a functional clock. These findings correlate with those of an earlier study which reported that night-time infection of flies with pathogenic bacteria produced better survival rates than that in the daytime [24]. Furthermore, *per*^01 ^flies were more susceptible to infection. Whether this is due to a disrupted sleep response or to other aspects of host defense is an interesting topic for future study.

### *Relish *is required for the sleep promoting effect of the immune response

**Figure 4:**
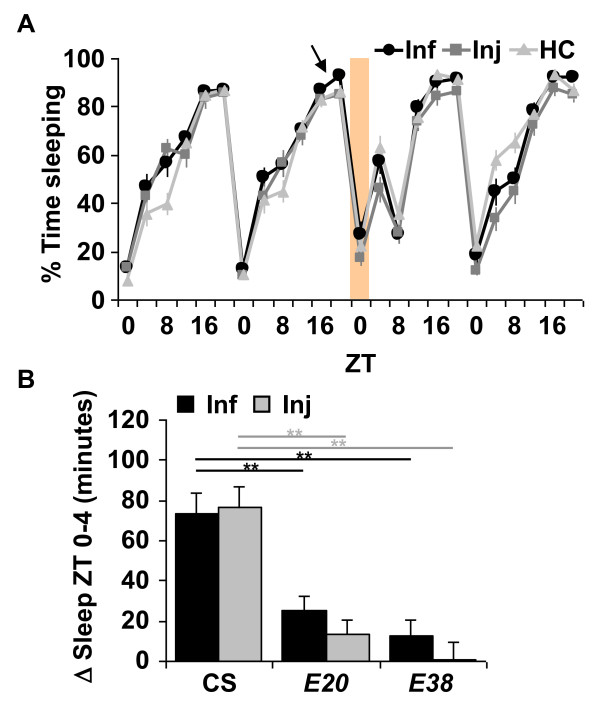
**NFκB *Relish *null mutants abolish the increase in sleep from ZT 0-4 after infection or aseptic injury**. (**A**) Representative results from treatment in *E20 *flies at ZT 18 (arrow). Data are plotted as described in Figure 1A. n = 14 for Inf, n = 16 for Inj and n = 16 for HC. (**B**) Mean ± SEM net changes in sleep from ZT 0-4 in CS, *E20 *and *E38 *flies that were treated at ZT 18. *p *< 0.0001, ANOVA in both Inf and Inj groups. ** *p *< 0.01; Tukey's post-hoc comparisons. n = 29-62 flies per group.

We next determined a role of the NFκB Relish in sleep that is triggered by an immune response. We focused on *Relish *because our previous study indicated that expression of its mRNA was very strongly affected by sleep deprivation, and that heterozygous mutants had reduced baseline, or spontaneous sleep [14]. Furthermore, as discussed above, Relish dependent gene expression is increased with both infection and injury (Figure 2B and [29]). Homozygous *Relish*^*E*20 ^(*E20*) null mutants are severely immunocompromised and die within a few days after infection with non-pathogenic bacteria such as *E. coli *[37]. *E20 *homozygous flies in a CS background [14] were subjected to infection with *E. coli *or aseptic injury, as described above. Flies infected at ZT 18 (Figure 4A) and at ZT 6 (Additional File 1: Table S1) did not increase sleep as compared to HC and instead, continued to maintain a relatively normal sleep pattern until they died. Sleep from ZT 0-4 in both infected and injured *E20 *flies showed no significant change as compared to HC (*p *> 0.1) but showed significantly reduced net changes in sleep as compared to CS flies (Figure 4B). To ensure the differences between wild-type and *E20 *flies was not attributable to handling, we compared sleep in handled control groups from ZT 0-4 during baseline (the morning before treatment) with sleep during the morning after treatment. No significant change in sleep was detected (data not shown; *p *> 0.5 for paired *t*-test comparisons within groups as well as *t*-test comparisons between CS and *E20*). Together, these observations indicate that the NFκB *Relish *is required for the increase in sleep during the immune response.

We next performed studies to confirm that this phenotype was attributable to a lesion in the *Relish *gene. First, another *Relish *null allele, *Relish*^*E*38 ^(*E38*) [37], was examined. Similar to the *E20 *flies, *E38 *mutants did not increase sleep in response to infection with *E. coli *or to injury (*p *> 0.4; Additional File 1: Table S1). The net change in sleep during the immune response was also significantly smaller as compared to CS flies (Figure 4B).

**Figure 5:**
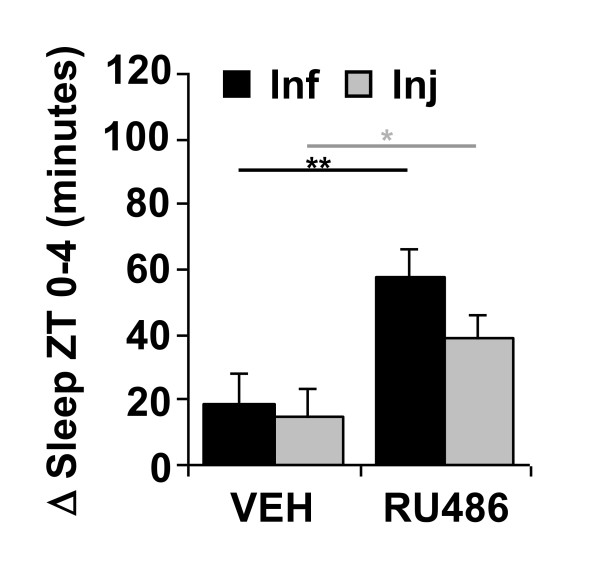
**Expression of *Relish *in fat bodies restores sleep induced by infection or injury in *E20 *mutants**. Mean ± SEM net changes in sleep from ZT 0-4 in *S*_1_*106-Gal4*/*UAS-Rel*; *E20 *flies. Flies were chronically exposed to vehicle (VEH) or RU486 and treated at ZT 18. ** *p *< 0.005 and *p < 0.05, *t*-test; n = 33-61 flies per group.

We next determined whether expressing a *UAS-Relish *transgene in fat bodies in *E20 *mutants could restore the increase in sleep during the immune response. Fat bodies are masses of adipose tissue located throughout the fly and are a major site of immune signaling and metabolism [18]. To circumvent the effects of genetic background, we used the RU486-dependent Gal4 driver *S*_1_*106 *[38], which expresses specifically in fat body. *S*_1_*106-Gal4*/*UAS-Rel*; *E20 *flies that were chronically fed RU486 showed a significant increase in sleep from ZT 0-4 after treatment at ZT 18 as compared to siblings of the same genotype that were fed a vehicle control (Figure 5; student's *t*-test, *p *< 0.005 for Inf and *p *< 0.05 for Inj). To determine whether the effect of *Relish *on sleep was specific to fat bodies, we used a pan-neuronal RU486-dependent Gal4 driver, *elav-GeneSwitch *(*elavGS*) [39] to express *UAS-Rel *in the central nervous system. Neither the RU486-fed nor vehicle control fed *elav-GS/UAS-Rel; E20 *flies showed a significant increase in morning sleep after infection (*p *> 0.1) or injury (*p *> 0.08) at ZT 18 (Additional File 5: Figure S4). Thus *UAS-Rel *restores sleep during an immune response in *E20 *mutants when expressed in fat bodies, but not in neurons.

These data demonstrate that *Relish *is necessary for the sleep promoting effect of the immune response and that its expression in fat body is sufficient for this effect. In our previous study, we reported that *Relish *also functioned from fat body to modulate baseline or spontaneous undisturbed sleep such that sleep was reduced in heterozygous *Relish *null mutants, but not affected in *Relish *homozygous mutants [14]. We now report that *Relish *homozygous mutants virtually abolish a sleep promoting effect of the immune response. Together these observations indicate that sleep induced by infection or injury is different from baseline sleep and that each is controlled by different mechanisms. Others have proposed that baseline and rebound sleep, which is sleep after prolonged wakefulness or sleep deprivation, are also controlled by different mechanisms [10,40]. For example, Hyperkinetic and shaker mutations strongly reduce daily sleep levels, but do not alter responses to sleep deprivation [10,41]. In contrast, a recent study described a hypomorph of the sleepless gene that minimally affected baseline sleep but had severe effects on sleep rebound [40]. Mutants of the clock gene, cycle, also reduced baseline sleep [42], but produced sex dimorphic effects on sleep rebound [42,43]. Thus the results described here indicate that the predominant role of Relish in sleep is during a recovery period after immune challenge.

As discussed earlier, injury and infection produce distinct effects on *κB-luc* activity such that the effect of infection increases reporter activity to a much greater extent (Figure 2B and Additional File 3: Figure S2B). Given the role of Relish in sleep induced by immune challenge, one may expect that the impact of infection on sleep would be greater than that during aseptic injury. This is clearly not the case, which suggests that a component other than NFκB limits the duration of the morning sleep response. One possibility is that the pressure to be awake at this time of day outweighs the pressure to sleep. However, the duration of this response was unchanged in *per*^01 ^mutants. In particular, daytime infection or injury in these flies produced an immediate increase in sleep that lasted approximately four hours, which is similar to the duration of the morning increase in sleep seen in wild type flies. This observation suggests that the circadian clock determines when the response occurs, but does not determine the duration of the effect on sleep. We propose that while *Relish *is necessary for inducing sleep during an immune response, a separate mechanism is involved in limiting the duration of sleep during a recovery period. This mechanism may involve neural circuits in the brain, which are known to regulate sleep (reviewed in [44]). Alternatively, negative regulatory factors that act directly on Relish target genes are also potential candidates that may be involved in this process [45].

Studies in mammals have shown that blocking IL-1 or TNFα cytokines also prevent increased sleep during immune challenge (reviewed in [46]), which is consistent with our current findings. NFκB p50 knockout mice also show reduced sleep responses to infection with influenza virus, but had enhanced responses to immune challenge with bacterial lipopolysaccharide [47]. Although Relish has similarity with p50, it is possible that different NFκB family members in both mammals and flies will have distinct roles in baseline sleep and in sleep induced by infection or injury.

## Conclusions

The observation that the immune response promotes sleep in Drosophila demonstrates an additional feature of insect sleep that is shared with that in mammals. The effect of infection and injury on sleep is gated by the circadian clock, such that the effect is restricted to the morning after treatment and is affected by the time-of-day of the treatment. Night-time injury or infection with Gram-negative bacteria produced stronger effects than equivalent manipulations in the daytime. This pattern is disrupted in the *per*^01 ^clock mutant, which responds by increasing sleep immediately after infection or injury. These findings correlate with the observation that survival rates were lower when flies were infected with Gram-negative pathogenic bacteria in the daytime than at nighttime [24]. We have also demonstrated that *Relish *is required for the sleep promoting effect of the immune response, and that its expression in fat body is sufficient for this process. Together, these findings suggest that adaptive sleep during an immune response involves an integration of signals from the peripheral immune system and the circadian clock. Further genetic dissection of this process will better our understanding of a function of sleep in recovery from immune challenge or injury.

## Methods

### Fly stocks

Flies were grown on standard agar, corn meal, malt extract, and soy flour medium with 1.84 mg/L tegosept. *Relish*^*E*20 ^(*E20*) mutants were isogenized to a CS background as described previously [14]. Briefly, the *ebony *marker was removed by recombination, and offspring were backcrossed into the CS background for at least four generations. The presence of the *E20 *mutation was confirmed using PCR. *κB-luc *transgenic flies were generated as described below. *w;S*_1_*106;Rel*^*E*20^, *w;elav-GS;Rel*^*E*20^, *w*^1118^, *per*^01 ^and *w*^1118^, *per*^01^*;; per *flies [35] were provided by Dr. Isaac Edery, Rutgers University. Other strains used were CS, *Relish*^*E*38^*(E38) *and *UAS-Rel*;*Rel*^*E*20^/*TM3, sb*.

### Behavioral assays

Sleep was measured as described previously [14]. All experiments were performed in females. Flies 1-3 days in age were loaded into glass activity tubes containing 5% sucrose and 2% agar medium, maintained in 12:12 light: dark cycles at 25°C, and activity was measured using the Trikinetics DAM2 system (Waltham, MA). For experiments in which flies were treated with drug, food contained 2% sucrose and 2% agar. Activity counts were collected every minute, and sleep defined as activity counts of zero for a minimum of 5 consecutive minutes [31]. Sleep parameters were analyzed using custom Matlab based software, Insomniac2, generously provided by Dr. Lesley Ashmore, University of Pennsylvania.

For induction of mifepristone (RU486; Sigma) dependent Gal4 drivers [38,39], 25 μM RU486 was added to standard fly food medium for chronic exposure throughout development. RU486 was diluted from a 10 mM stock in 80% ethanol. Equivalent dilutions were made with 80% ethanol for the vehicle control groups. Adult flies were exposed to 500 μM RU486, or equivalent control vehicle dilution, in sucrose/agar medium used in the activity tubes for behavioural experiments.

### Infection and Injury

Infections were conducted by injecting flies with *E. coli *or *P. aeruginosa *using small glass pipettes (tip diameter ~50 μm). Bacteria were grown to saturating concentrations (OD_600 _= 0.5 - 1.0) in LB medium containing 50 μg/ml ampicillin (for *E. coli*) or 50 μg/ml gentamycin (for *P. aeruginosa*) and then diluted in phosphate buffered saline (PBS) and food coloring solution. The final concentrations for bacteria were OD_600 _of 0.1 for *E. coli *and OD_600 _of 1 × 10^-4 ^for *P. aeruginosa*. A second group of flies (injured groups, or Inj) was subjected to injections of dilutions of LB broth (with indicated antibiotics) in PBS/food coloring solution. A third group (handled control, or HC) was subjected to the same handling, but not injected.

For treatment in both LD and DD cycles, activity monitors containing flies were removed from incubators. Individual flies were then removed from each glass activity tube, placed onto a CO_2 _pad for anesthetization, and were infected or injured. Handled control flies were CO_2 _anesthetized for the same duration as the infected and injured groups. Flies that were treated during night-time hours or in DD were typically exposed to a 1 hour light pulse. The time of treatment in constant dark conditions corresponded to times when light pulses produce minimal phase shifts in locomotor activity rhythms [48].

### Luciferase reporter assay

Transgenic flies (*κB-luc*) that express a luciferase reporter (*cecκB-luc*) were generated as follows: The *cecκB-luc *reporter plasmid was constructed by inserting into a pGL3 basic luciferase vector (Promega), 8 direct repeats of NFκB binding sequences (5'-ATCGGGGATTTTTGCAGAGAAAA-3') derived from the *cecropin A1 *promoter region [49] followed by a *Drosophila *heat shock protein (Hsp70Bb) basal promoter. The *cecκB-luc *construct was then subcloned into a pCaSpeR4 backbone. Transgenic flies were then generated in a *w*^1118 ^background (BestGene Inc., CA).

*κB-luc *flies 1-3 days in age maintained in 12:12 light: dark cycles were loaded into vials containing 5% sucrose, 2% agar medium and entrained for another 2 days. 2 days later flies were transferred individually to a 96-well plate containing 2 mM luciferin (the substrate of luciferase; Gold Biotechnology Inc.), 2% sucrose and 1% agar medium. Flies were then subjected to infection with *E. coli *or injury, as described above, at ZT 18 the next day. Luciferase activity in living flies was measured immediately before treatment at ZT 18 and after treatment (ZT 0 and ZT 4) with a Fusion Universal Microplate Analyzer (Packard). A control group was subjected to CO_2 _anesthesia for same duration as the other groups, but was not injured or infected.

A previous study demonstrated that a similar construct was sensitive to the NFκBs Dif and Dorsal in an insect cell culture system [49]. We determined whether the *κB-luc *reporter was also sensitive to Relish activity in living flies. *κB-luc *was expressed in *Relish*^*E*20 ^homozygous mutants and in *E20/TM3, Sb *heterozygous siblings. Flies were infected with *E. coli *at night- time, ZT 18, and luciferase reporter activity was measured at ZT 18 prior to infection, ZT 0, and ZT 4, as described above. A significant induction of *κB-luc *was detected in both groups of flies (*p *< .0001 ANOVA) such that *κB-luc *activity was significantly higher at ZT 4 as compared to baseline at ZT 18 and ZT 0 (*p *< 0.01, Tukey's post-hoc comparisons; see Additional File 6: Figure S5). However, the signal from *E20/E20 *homozygous flies was significantly lower than the heterozygous siblings at baseline, ZT 18 (*p *< .0001; student's *t*-test), and values from the *E20/E20 *flies at ZT 4 did not differ significantly from the baseline values of the *E20/TM3sb *flies (*p *< .066). Although we detected an induction of *κB-luc *during infection in the *E20/E20 *flies, the level of induction did not surpass the baseline values in the control siblings. These data indicate that the *κB-luc *signal is highly sensitive to Relish activity during infection with *E. coli*.

### Statistical analysis

To analyze changes in sleep during the immune response, a statistical algorithm was developed in collaboration with Dr. Minge Xie and Chingray Yu in the Department of Statistics, Rutgers University, to make *t*-test comparisons between the amount of sleep at a given time points for treated and handled control groups. This algorithm is based on the simple formula:

where I = infected or injured group, T_0 _= sleep time from ZT 0-4 before treatment, T_1 _= sleep time from ZT 0-4 post-treatment, C = handled control and n_*C *_= number of flies in the corresponding control group. This is a conservative approach that corrects for arbitrary differences in baseline sleep that may occur between conditions and is the basis for calculating a *t*-statistic for comparing sleep before and after treatment. The net changes in sleep after treatment were determined by formula [i]. *t*-tests were performed using custom software (available upon request) written in R version 2.3.1, downloaded from http://www.r-project.org/. Using this approach, sleep during the immune response was initially analyzed using a range of increments (ZT 0-1, 0-2, 0-4, 0-6 and 0-8). We found that bacterial infection or aseptic injury consistently produced the most robust effect on sleep from ZT 0-4. Values reported in Additional File 1: Table S1 and in Figures 1 and 3 for treatments in the daytime, ZT 0 and ZT 6, correspond to the ZT 0-4 time point 24 h and 18 h, respectively, after treatment. The immediate response at ZT 0-4 elicited by treatment at ZT 0 is discussed in the main text and is reported in Additional File 4: Figure S3.

Multi-group comparisons between mean net changes in sleep were performed by one-way ANOVA http://statpages.org/ followed by Tukey's post-hoc comparison through online software from the Department of Obstetrics and Gynecology, The Chinese University of Hong Kong http://department.obg.cuhk.edu.hk/researchsupport/statstesthome.asp. For the luciferase reporter assays, within group comparisons using ANOVA were performed to determine whether the signal was affected after treatment in each group (Inf, Inj, or HC). Comparisons were also made at each time point using student's *t*-test to determine differences from the handled control group.

## Authors' contributions

T-HK and DHP performed and analyzed experiments to characterize sleep during the immune response in both wild type and mutant flies. T-HK generated the *κB-luc *construct, performed experiments in *κB-luc *flies, and determined the effects of *UAS-Relish *on restoring sleep in *Relish *mutants. ZB performed experiments conducted in DD and analyzed the effect of *P. aeruginosa *on sleep. JAW designed and supervised experiments. T-HK and JAW wrote the manuscript. All authors read and approved the final manuscript.

## Supplementary Material

Additional file 1 **Table S1.** Sleep from ZT 0-4 following infection or injury at indicated times. Mean ± SEM values for minutes sleep during the 4 hour morning period following treatment. HC = handled control, Inj = Injury, and Inf = Infection with *E. coli*. *p *values are derived from *t*-test comparisons between the change in sleep before (BL = baseline) and after (PT = post-treatment) treatment with that from the corresponding HC group (using formula [i], see Methods). For flies treated at ZT 0, the morning ZT 0-4 time is the day 24 h *after *infection or injury. See text for discussion of immediate effects of infection and injury on sleep. Click here for file

Additional file 2 **Figure S1.** Infection and injury do not affect locomotor ability of flies. Activity counts per waking minute (activity rate) of infected, injured and HC groups during the morning after treatment at ZT 18. *p < 0.05, Tukey's post-hoc comparison; n = 42-61 flies per group. Click here for file

Additional file 3 **Figure S2.** Infection with *P. aeruginosa *promotes morning sleep similar to injury. (A) Representative experiment in CS flies that were infected with *P. aeruginosa *(Inf) or injured (Inj) at ZT 18 (arrow). Data are plotted as described in Figure 1A. n = 10 for Inf, n = 14 for Inj and n = 13 for HC. (B) Mean ± SEM luciferase activity (arbitrary units) was measured at indicated ZT times when *κB-luc *flies were infected with *P. aeruginosa *or injured at ZT 18. ***p < 0.0005, **p < 0.005 and *p < 0.05, student's *t*-test compared to HC at corresponding time points. n = 28-48 flies for each group. Click here for file

Additional file 4 **Figure S3.***per*^01 ^flies increase sleep immediately after infection with *E. coli *or aseptic injury. (A) *per*^01 ^flies were treated at indicated time points, and mean ± SEM net changes in sleep are plotted for the 4 h period immediately after treatment. ANOVA, *p *< 0.0001 in Inj group. ** *p *< 0.01 and * *p *< 0.05, Tukey's post-hoc comparison. n = 13-16 flies per group. (B) Mean ± SEM net changes in sleep in *per*^01^*;; per *flies for the 4 h period immediately after treatment at each of four indicated time points. ANOVA, *p *< 0.0001 in both Inf and Inj groups.** *p *< 0.01 and * *p *< 0.05, Tukey's post-hoc comparison. n = 22-45 flies per group. Click here for file

Additional file 5 **Figure S4.** Expression of *Relish *in neurons does not restore sleep during infection or injury in *E20 *mutants. Mean ± SEM net changes in sleep from ZT 0-4 in *elav*^*GS*^/*UAS-Rel*; *E20 *flies. Flies were chronically fed vehicle (VEH) or RU486 and treated at ZT 18. n = 16-48 flies per group. Click here for file

Additional file 6 **Figure S5.***κB-luc *reporter activity is sensitive to NFκB Relish. Mean ± SEM luciferase activity (arbitrary units) was measured at indicated ZT times when flies were infected with *E. coli *at ZT 18. Infected flies included *κB-luc *expressed in siblings carrying one copy of *Rel*^*E*20 ^(*κB-luc;;Rel*^*E*20^/*TM3, Sb*; n = 16) or two copies of *Rel*^*E*20 ^(*κB-luc;;Rel*^*E*20^; n = 32). **p < 0.0001, *t*-test comparison between two genotypes at indicated time points. Click here for file
