# Supplementary material for: Sleep triggered by an immune response in Drosophila is regulated by the circadian clock and requires the NFκB Relish
Source: BMC Neurosci. 2010 Feb 9;11:17. doi: 10.1186/1471-2202-11-17 (PMC2831041; doi:10.1186/1471-2202-11-17)
Supplement: Additional file 1 — Table S1. Sleep from ZT 0-4 following infection or injury at indicated times. Mean ± SEM values for minutes sleep during the 4 hour morning period following treatment. HC = handled control, Inj = Injury, and Inf = Infection with E. coli. p values are derived from t-test comparisons between the change in sleep before (BL = baseline) and after (PT = post-treatment) treatment with that from the corresponding HC group (using formula [i], see Methods). For flies treated at ZT 0, the morning ZT 0-4 time is the day 24 h after infection or injury. See text for discussion of immediate effects of infection and injury on sleep. [file 1471-2202-11-17-S1.PDF]

		ZT 0				ZT 6				ZT 12				ZT 18			
**Genotype**		n	BL	PT	*p*	n	BL	PT	*p*	n	BL	PT	*p*	n	BL	PT	*p*
CS	HC	31	35.6 ±6.4	23.8 ±3.3		32	33.4 ±5.5	24.0 ±3.9		32	67.5 ±7.1	48.4 ±9.1		59	34.6 ±3.2	33.9 ±4.5	
	Inj	31	24.4 ±4.7	28.5 ±4.9	.269	31	39.7 ±7.0	62.6 ±10.3	**.020**	29	52.2 ±9.4	126.9 ±12.1	**.0002**	61	40.4 ±4.0	116.8 ±9.5	**3.2E-10**
	Inf	30	26.7 ±4.9	36.4 ±8.3	.140	29	49.5 ±7.5	62.8 ±11.7	.103	26	47.2 ±6.7	124.9 ±14.6	**.0002**	42	40.0 ±5.2	109.4 ±10.8	**2.77E-7**
Relish^{E20}	HC					16	41.8 ±8.3	37.7 ±11.0						62	35.7 ±4.9	38.1 ±5.9	
	Inj					16	28.3 ±13.7	41.7 ±10.6	.517					62	37.5 ±4.8	53.7 ±6.7	.368
	Inf					15	16.1 ±6.8	22.0 ±5.8	.717					43	37.3 ±6.4	65.6 ±7.5	.127
Relish^{E38}	HC													31	31.6 ±7.3	58.1 ±8.2	
	Inj													29	45.1 ±9.5	75.0 ±8.6	.879
	Inf													29	34.3 ±5.8	76.0 ±7.6	.491
per^{01}	HC	14	8.3 ±1.9	5.3 ±1.9		14	25.7 ±5.8	8.6 ±2.3		16	16.9 ±3.3	8.1 ±3.1		13	23.5 ±5.4	24.2 ±6.4	
	Inj	15	9.7 ±3.3	9.3 ±2.9	.604	15	11.0 ±4.0	11.0 ±3.8	.156	15	22.7 ±9.3	18.3 ±3.8	.628	16	25.6 ±5.9	41.6 ±12.9	.333
	Inf	15	7.3 ±2.3	6.0 ±1.7	.745	16	17.5 ±4.8	13.8 ±7.1	.257	16	17.2 ±5.5	15.6 ±6.5	.424	14	32.1 ±10.7	46.1 ±9.4	.415
per^{01}*;; per*	HC	28	59.1 ±9.8	47.0 ±6.8		32	50.2 ±5.3	23.2 ±3.3		30	62.1 ±7.0	48.7 ±5.9		45	58.6 ±6.9	71.0 ±7.5	
	Inj	28	54.1 ±6.2	55.1 ±7.4	.580	32	52.8 ±5.7	51.0 ±10.0	**.046**	30	54.2 ±5.5	78.4 ±6.8	**.042**	37	50.3 ±6.7	124.6 ±9.9	**.0045**
	Inf	22	60.3 ±10.6	60.3 ±9.3	.634	30	57.4 ±6.5	40.9 ±6.4	.406	27	51.6 ±7.6	64.1 ±8.0	.168	42	52.5 ±6.3	106.5 ±9.0	**.045**

Genotype		n	BL	PT	*p*	n	BL	PT	*p*	n	BL	PT	*p*	n	BL	PT	*p*
$S_1$106/UAS-Rel; Relish[E20] (Vehicle)	HC													51	31.0 ±3.9	43.2 ±4.9	
	Inj													53	21.0 ±5.8	37.0 ±9.1	.209
	Inf													33	22.5 ±3.0	51.1 ±9.9	.247
$S_1$106/UAS-Rel; Relish[E20] (RU486)	HC													61	52.7 ±4.9	52.6 ±6.7	
	Inj													49	61.7 ±5.2	100.5 ±9.0	**.015**
	Inf													36	52.3 ±7.2	109.9 ±10.6	**.0014**
elavGS/UAS-Rel; Relish[E20] (Vehicle)	HC													47	76.2 ±8.5	77.1 ±7.7	
	Inj													48	84.7 ±7.4	124.2 ±8.5	.090
	Inf													23	97.7 ±12.1	136.8 ±11.4	.178
elavGS /UAS-Rel; Relish[E20] (RU486)	HC													34	48.8 ±7.7	53.2 ±8.0	
	Inj													32	40.7 ±6.7	71.9 ±10.1	.229
	Inf													16	72.3 ±16.8	114.3 ±19.4	.181
