# Supplementary figures and images for: Sleep triggered by an immune response in Drosophila is regulated by the circadian clock and requires the NFκB Relish
Source: BMC Neurosci. 2010 Feb 9;11:17. doi: 10.1186/1471-2202-11-17 (PMC2831041; doi:10.1186/1471-2202-11-17)

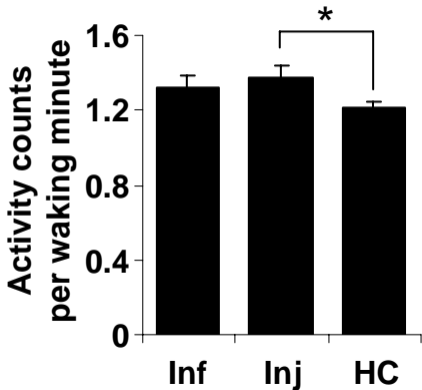

Supplement: Additional file 2 — Figure S1. Infection and injury do not affect locomotor ability of flies. Activity counts per waking minute (activity rate) of infected, injured and HC groups during the morning after treatment at ZT 18. *p < 0.05, Tukey's post-hoc comparison; n = 42-61 flies per group. [file 1471-2202-11-17-S2.PDF]

**A**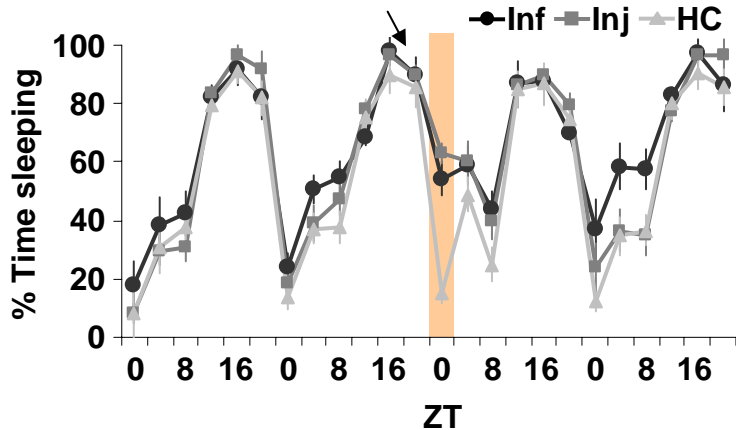**B**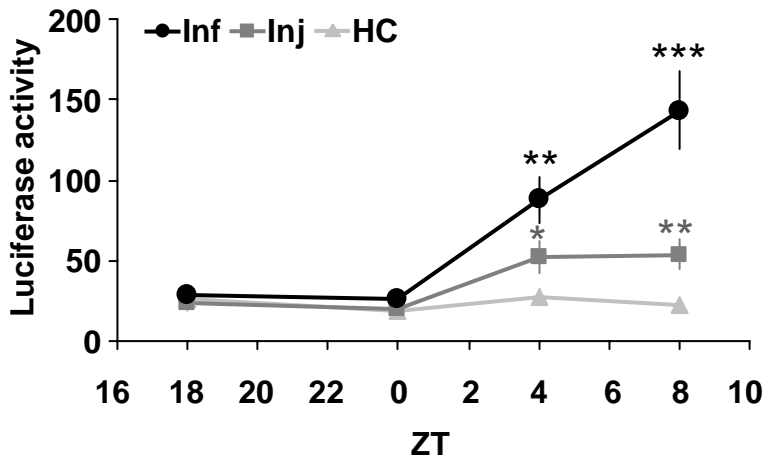

Supplement: Additional file 3 — Figure S2. Infection with P. aeruginosa promotes morning sleep similar to injury. (A) Representative experiment in CS flies that were infected with P. aeruginosa (Inf) or injured (Inj) at ZT 18 (arrow). Data are plotted as described in Figure 1A. n = 10 for Inf, n = 14 for Inj and n = 13 for HC. (B) Mean ± SEM luciferase activity (arbitrary units) was measured at indicated ZT times when κB-luc flies were infected with P. aeruginosa or injured at ZT 18. ***p < 0.0005, **p < 0.005 and *p < 0.05, student's t-test compared to HC at corresponding time points. n = 28-48 flies for each group. [file 1471-2202-11-17-S3.PDF]

**A**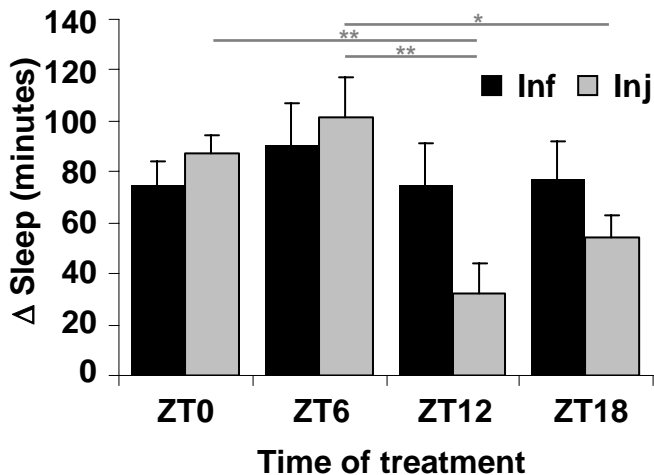**B**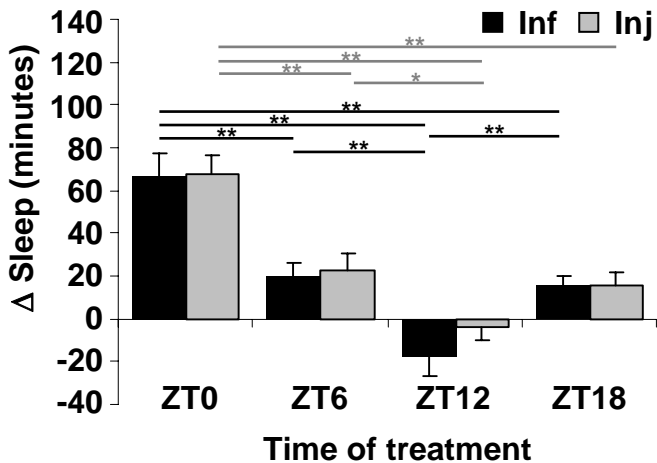

Supplement: Additional file 4 — Figure S3.per01 flies increase sleep immediately after infection with E. coli or aseptic injury. (A) per01 flies were treated at indicated time points, and mean ± SEM net changes in sleep are plotted for the 4 h period immediately after treatment. ANOVA, p < 0.0001 in Inj group. ** p < 0.01 and * p < 0.05, Tukey's post-hoc comparison. n = 13-16 flies per group. (B) Mean ± SEM net changes in sleep in per01;; per flies for the 4 h period immediately after treatment at each of four indicated time points. ANOVA, p < 0.0001 in both Inf and Inj groups.** p < 0.01 and * p < 0.05, Tukey's post-hoc comparison. n = 22-45 flies per group. [file 1471-2202-11-17-S4.PDF]

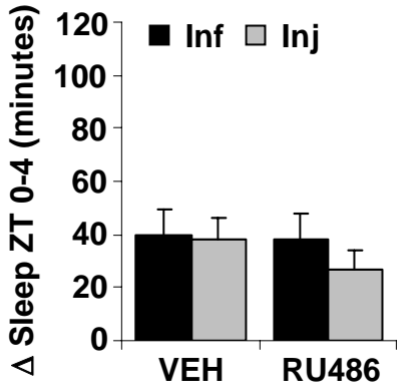

Supplement: Additional file 5 — Figure S4. Expression of Relish in neurons does not restore sleep during infection or injury in E20 mutants. Mean ± SEM net changes in sleep from ZT 0-4 in elavGS/UAS-Rel; E20 flies. Flies were chronically fed vehicle (VEH) or RU486 and treated at ZT 18. n = 16-48 flies per group. [file 1471-2202-11-17-S5.PDF]

Luciferase activity

400

■  $\kappa B\_luc;;Rel^{E20}/TM3, Sb$

▲  $\kappa B\_luc;;Rel^{E20}$

200

0

16

18

20

22

0

2

4

6

ZT

\*\*

\*\*

\*\*

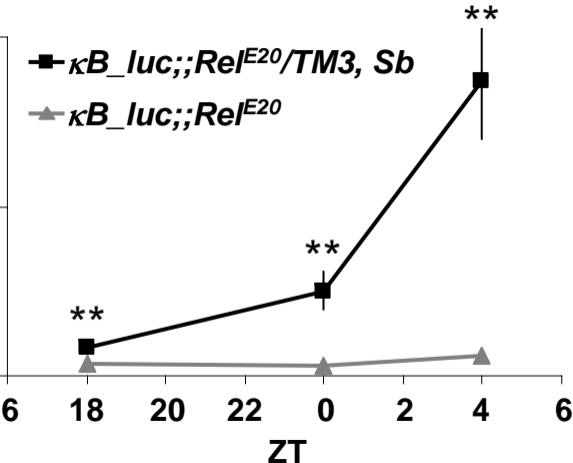

Supplement: Additional file 6 — Figure S5.κB-luc reporter activity is sensitive to NFκB Relish. Mean ± SEM luciferase activity (arbitrary units) was measured at indicated ZT times when flies were infected with E. coli at ZT 18. Infected flies included κB-luc expressed in siblings carrying one copy of RelE20 (κB-luc;;RelE20/TM3, Sb; n = 16) or two copies of RelE20 (κB-luc;;RelE20; n = 32). **p < 0.0001, t-test comparison between two genotypes at indicated time points. [file 1471-2202-11-17-S6.PDF]
